# *N*-Benzyl­propan-2-aminium chloride

**DOI:** 10.1107/S1600536810006707

**Published:** 2010-02-27

**Authors:** Mehrdad Pourayoubi, Monireh Negari

**Affiliations:** a Department of Chemistry, Ferdowsi University of Mashhad, Mashhad 91779, Iran

## Abstract

In the crystal structure of title salt, C_10_H_16_N^+^·Cl^−^, the amino H atoms are involved in inter­molecular N—H⋯Cl hydrogen bonding, generating a zigzag chain propagating in [100].

## Related literature

For related structures, see: Pourayoubi & Sabbaghi (2007 ▶); Yazdanbakhsh & Sabbaghi (2007 ▶). 
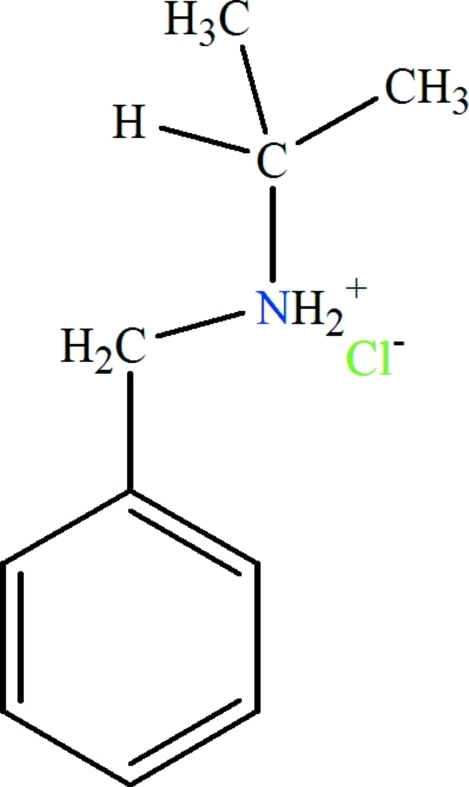


## Experimental

### 

#### Crystal data

C_10_H_16_N^+^·Cl^−^
*M* *_r_* = 185.69
Orthorhombic, 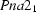

*a* = 9.9666 (6) Å
*b* = 18.0379 (11) Å
*c* = 5.7307 (4) Å
*V* = 1030.25 (11) Å^3^
*Z* = 4
Mo *K*α radiation
μ = 0.32 mm^−1^
*T* = 100 K
0.50 × 0.40 × 0.30 mm

#### Data collection

Bruker APEXII CCD diffractometer
Absorption correction: multi-scan (*SADABS*; Bruker, 2005 ▶) *T*_min_ = 0.818, *T*_max_ = 0.910
11694 measured reflections
2720 independent reflections
2662 reflections with *I* > 2σ(*I*)
*R*_int_ = 0.023

#### Refinement

*R*[*F* ^2^ > 2σ(*F* ^2^)] = 0.021
*wR*(*F* ^2^) = 0.057
*S* = 1.07
2720 reflections
119 parameters
1 restraint
H atoms treated by a mixture of independent and constrained refinement
Δρ_max_ = 0.25 e Å^−3^
Δρ_min_ = −0.15 e Å^−3^
Absolute structure: Flack (1983 ▶), 1229 Friedel pairs
Flack parameter: −0.02 (4)

### 

Data collection: *APEX2* (Bruker, 2005 ▶); cell refinement: *SAINT* (Bruker, 2005 ▶); data reduction: *SAINT*; program(s) used to solve structure: *SHELXS97* (Sheldrick, 2008 ▶); program(s) used to refine structure: *SHELXL97* (Sheldrick, 2008 ▶); molecular graphics: *SHELXTL* (Sheldrick, 2008 ▶); software used to prepare material for publication: *SHELXTL*.

## Supplementary Material

Crystal structure: contains datablocks I, global. DOI: 10.1107/S1600536810006707/ng2732sup1.cif

Structure factors: contains datablocks I. DOI: 10.1107/S1600536810006707/ng2732Isup2.hkl

Additional supplementary materials:  crystallographic information; 3D view; checkCIF report

## Figures and Tables

**Table 1:** Hydrogen-bond geometry (Å, °)

*D*—H⋯*A*	*D*—H	H⋯*A*	*D*⋯*A*	*D*—H⋯*A*
N1—H1⋯Cl1	0.853 (13)	2.288 (13)	3.1296 (9)	168.8 (11)
N1—H2⋯Cl1^i^	0.877 (14)	2.255 (14)	3.1257 (9)	171.9 (13)

Symmetry code: (i) 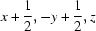.

**Fig. 1.**
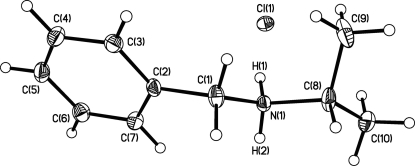
The molecular structure of the title salt, showing the atom-numbering scheme. Displacement ellipsoids are drawn at the 50 % probability level.

**Fig. 2.**
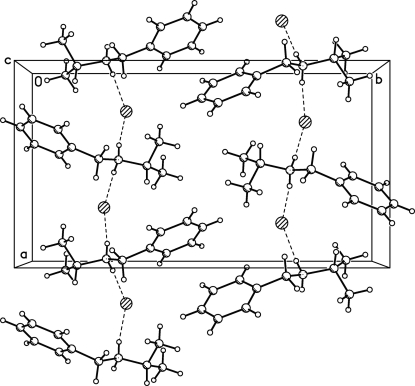
Fragment of crystal packing (projection along c crystal axis), the hydrogen bonds are shown by dash line.

## supplementary crystallographic information

### Comment

In the previous works, the structure determination of [NH_2_(CH_2_C_6_H_5_)(CH(CH_3_)_2_)][CCl_3_C(O)NHP(O)(O)(OCH_3_)] (Pourayoubi & Sabbaghi, 2007) and [NH_2_(CH_2_C_6_H_5_)(CH(CH_3_)_2_)] [CF_3_C(O)NHP(O)(O)(N(CH_2_C_6_H_5_)(CH(CH_3_)_2_)] (Yazdanbakhsh & Sabbaghi, 2007) have been investigated; we report here on the crystal structure of title compound, the chloride salt of *N*-benzyl-2-propanaminium cation (Fig. 1). Both hydrogen atoms of NH_2_ groups are involved in intermolecular N—H···Cl hydrogen bonding with neighbouring Cl^-^ anions [N1···Cl1 = 3.1296 (9) Å, N1···Cl2 = 3.1257 (9) Å] into an extended 1-D zigzag chain (Fig. 2).

### Experimental

The title compound is a by-product of the preparation of P(O)[OC_6_H_5_][N(CH_2_C_6_H_5_)(CH(CH_3_)_2_)]_2_ [from the reaction between P(O)[OC_6_H_5_]Cl_2_ and NH(CH_2_C_6_H_5_)(CH(CH_3_)_2_), with 1:4 mole ratio] which is crystallized in CH_3_C(O)CH_3_.

### Refinement

The H atoms of the NH2 group were located from the difference Fourier synthesis and refined isotropically, no restraints were used. Finally, the geometrical and thermal parameters obtained for these H-atoms, as well as parameters of the hydrogen bonds for these H-atoms included, were rather realistic. The H(C) atom positions were calculated and refined in isotropic approximation in riding model with the Uiso(H) parameters equal to 1.2 Ueq(Ci), for methyl groups equal to 1.5 Ueq(Cii), where U(Ci) and U(Cii) are respectively the equivalent thermal parameters of the carbon atoms to which corresponding H atoms are bonded.

### Crystal data

**Table:**

C_10_H_16_N^+^·Cl^−^	*F*(000) = 400
*M**_r_* = 185.69	*D*_x_ = 1.197 Mg m^−^^3^
Orthorhombic, *P**n**a*2_1_	Mo *K*α radiation, λ = 0.71073 Å
Hall symbol: P 2c -2n	Cell parameters from 8636 reflections
*a* = 9.9666 (6) Å	θ = 2.3–34.0°
*b* = 18.0379 (11) Å	µ = 0.32 mm^−^^1^
*c* = 5.7307 (4) Å	*T* = 100 K
*V* = 1030.25 (11) Å^3^	Prism, colourless
*Z* = 4	0.50 × 0.40 × 0.30 mm

### Data collection

**Table:**

Bruker APEXII CCD diffractometer	2720 independent reflections
Radiation source: fine-focus sealed tube	2662 reflections with *I* > 2σ(*I*)
graphite	*R*_int_ = 0.023
φ and ω scans	θ_max_ = 29.0°, θ_min_ = 2.3°
Absorption correction: multi-scan (*SADABS*; Bruker, 2005)	*h* = −13→13
*T*_min_ = 0.818, *T*_max_ = 0.910	*k* = −24→24
11694 measured reflections	*l* = −7→7

### Refinement

**Table:**

Refinement on *F*^2^	Secondary atom site location: difference Fourier map
Least-squares matrix: full	Hydrogen site location: difference Fourier map
*R*[*F*^2^ > 2σ(*F*^2^)] = 0.021	H atoms treated by a mixture of independent and constrained refinement
*wR*(*F*^2^) = 0.057	*w* = 1/[σ^2^(*F*_o_^2^) + (0.0336*P*)^2^ + 0.1229*P*] where *P* = (*F*_o_^2^ + 2*F*_c_^2^)/3
*S* = 1.07	(Δ/σ)_max_ = 0.001
2720 reflections	Δρ_max_ = 0.25 e Å^−^^3^
119 parameters	Δρ_min_ = −0.15 e Å^−^^3^
1 restraint	Absolute structure: Flack (1983), 1229 Friedel pairs
Primary atom site location: structure-invariant direct methods	Flack parameter: −0.02 (4)

### Special details

**Table:**

Geometry. All esds (except the esd in the dihedral angle between two l.s. planes) are estimated using the full covariance matrix. The cell esds are taken into account individually in the estimation of esds in distances, angles and torsion angles; correlations between esds in cell parameters are only used when they are defined by crystal symmetry. An approximate (isotropic) treatment of cell esds is used for estimating esds involving l.s. planes.
Refinement. Refinement of *F*^2^ against ALL reflections. The weighted *R*-factor wR and goodness of fit *S* are based on *F*^2^, conventional *R*-factors *R* are based on *F*, with *F* set to zero for negative *F*^2^. The threshold expression of *F*^2^ > σ(*F*^2^) is used only for calculating *R*-factors(gt) etc. and is not relevant to the choice of reflections for refinement. *R*-factors based on *F*^2^ are statistically about twice as large as those based on *F*, and *R*- factors based on ALL data will be even larger.

### Fractional atomic coordinates and isotropic or equivalent isotropic displacement parameters (Å^2^)

**Table:**

	*x*	*y*	*z*	*U*_iso_*/*U*_eq_	
Cl1	0.212804 (19)	0.282008 (11)	0.24289 (5)	0.01590 (6)	
N1	0.47016 (8)	0.26697 (4)	0.55218 (15)	0.01274 (15)	
H1	0.3950 (14)	0.2666 (7)	0.481 (2)	0.013 (3)*	
H2	0.5322 (12)	0.2517 (7)	0.455 (3)	0.014 (3)*	
C1	0.47024 (10)	0.21793 (5)	0.7630 (2)	0.01667 (19)	
H1A	0.4181	0.2423	0.8884	0.020*	
H1B	0.5636	0.2123	0.8190	0.020*	
C2	0.41198 (9)	0.14208 (5)	0.71879 (19)	0.01422 (17)	
C3	0.32866 (10)	0.11207 (6)	0.88949 (19)	0.01721 (19)	
H3A	0.3064	0.1405	1.0235	0.021*	
C4	0.27777 (10)	0.04055 (6)	0.8647 (2)	0.0205 (2)	
H4A	0.2218	0.0202	0.9826	0.025*	
C5	0.30877 (11)	−0.00100 (6)	0.6679 (2)	0.0192 (2)	
H5A	0.2743	−0.0498	0.6512	0.023*	
C6	0.39045 (10)	0.02910 (5)	0.49499 (19)	0.0188 (2)	
H6A	0.4105	0.0010	0.3591	0.023*	
C7	0.44277 (10)	0.10012 (5)	0.52059 (18)	0.01675 (18)	
H7A	0.4995	0.1202	0.4033	0.020*	
C8	0.50162 (11)	0.34699 (5)	0.6084 (2)	0.0195 (2)	
H8A	0.5825	0.3487	0.7116	0.023*	
C9	0.38428 (13)	0.38228 (5)	0.7366 (2)	0.0281 (2)	
H9A	0.3631	0.3530	0.8758	0.042*	
H9B	0.3059	0.3837	0.6335	0.042*	
H9C	0.4083	0.4329	0.7830	0.042*	
C10	0.53322 (12)	0.38753 (6)	0.3829 (2)	0.0256 (2)	
H10A	0.6057	0.3617	0.3007	0.038*	
H10B	0.5613	0.4384	0.4184	0.038*	
H10C	0.4530	0.3887	0.2840	0.038*	

### Atomic displacement parameters (Å^2^)

**Table:**

	*U*^11^	*U*^22^	*U*^33^	*U*^12^	*U*^13^	*U*^23^
Cl1	0.01334 (10)	0.01922 (10)	0.01515 (10)	0.00008 (7)	−0.00118 (9)	0.00016 (10)
N1	0.0131 (4)	0.0111 (3)	0.0140 (4)	−0.0004 (3)	−0.0017 (3)	0.0011 (3)
C1	0.0237 (4)	0.0127 (4)	0.0136 (5)	−0.0013 (3)	−0.0044 (4)	0.0014 (3)
C2	0.0155 (4)	0.0114 (4)	0.0157 (4)	0.0011 (3)	−0.0018 (4)	0.0034 (4)
C3	0.0181 (4)	0.0173 (4)	0.0162 (4)	0.0020 (3)	0.0013 (4)	0.0012 (4)
C4	0.0197 (5)	0.0199 (5)	0.0221 (6)	−0.0024 (4)	0.0022 (4)	0.0062 (4)
C5	0.0189 (4)	0.0143 (4)	0.0243 (5)	−0.0027 (4)	−0.0041 (4)	0.0029 (3)
C6	0.0209 (4)	0.0163 (4)	0.0192 (5)	−0.0003 (4)	−0.0018 (4)	−0.0021 (4)
C7	0.0187 (4)	0.0154 (4)	0.0162 (4)	−0.0016 (3)	0.0008 (4)	0.0003 (4)
C8	0.0234 (5)	0.0113 (4)	0.0238 (5)	−0.0048 (4)	−0.0083 (4)	0.0010 (4)
C9	0.0512 (6)	0.0125 (4)	0.0205 (5)	0.0038 (4)	0.0042 (6)	−0.0007 (5)
C10	0.0241 (5)	0.0171 (5)	0.0355 (6)	−0.0021 (4)	0.0051 (5)	0.0084 (4)

### Geometric parameters (Å, °)

**Table:**

N1—C1	1.4974 (13)	C5—H5A	0.9500
N1—C8	1.5118 (13)	C6—C7	1.3910 (14)
N1—H1	0.853 (14)	C6—H6A	0.9500
N1—H2	0.877 (14)	C7—H7A	0.9500
C1—C2	1.5076 (12)	C8—C10	1.5178 (16)
C1—H1A	0.9900	C8—C9	1.5207 (17)
C1—H1B	0.9900	C8—H8A	1.0000
C2—C3	1.3927 (14)	C9—H9A	0.9800
C2—C7	1.3990 (14)	C9—H9B	0.9800
C3—C4	1.3934 (15)	C9—H9C	0.9800
C3—H3A	0.9500	C10—H10A	0.9800
C4—C5	1.3892 (16)	C10—H10B	0.9800
C4—H4A	0.9500	C10—H10C	0.9800
C5—C6	1.3925 (15)		
			
C1—N1—C8	113.08 (8)	C7—C6—C5	120.21 (10)
C1—N1—H1	112.4 (9)	C7—C6—H6A	119.9
C8—N1—H1	107.0 (8)	C5—C6—H6A	119.9
C1—N1—H2	109.1 (9)	C6—C7—C2	120.11 (9)
C8—N1—H2	106.8 (8)	C6—C7—H7A	119.9
H1—N1—H2	108.2 (14)	C2—C7—H7A	119.9
N1—C1—C2	113.60 (9)	N1—C8—C10	108.76 (9)
N1—C1—H1A	108.8	N1—C8—C9	110.07 (8)
C2—C1—H1A	108.8	C10—C8—C9	111.67 (9)
N1—C1—H1B	108.8	N1—C8—H8A	108.8
C2—C1—H1B	108.8	C10—C8—H8A	108.8
H1A—C1—H1B	107.7	C9—C8—H8A	108.8
C3—C2—C7	119.39 (8)	C8—C9—H9A	109.5
C3—C2—C1	117.67 (9)	C8—C9—H9B	109.5
C7—C2—C1	122.88 (9)	H9A—C9—H9B	109.5
C2—C3—C4	120.36 (9)	C8—C9—H9C	109.5
C2—C3—H3A	119.8	H9A—C9—H9C	109.5
C4—C3—H3A	119.8	H9B—C9—H9C	109.5
C5—C4—C3	120.08 (9)	C8—C10—H10A	109.5
C5—C4—H4A	120.0	C8—C10—H10B	109.5
C3—C4—H4A	120.0	H10A—C10—H10B	109.5
C4—C5—C6	119.83 (9)	C8—C10—H10C	109.5
C4—C5—H5A	120.1	H10A—C10—H10C	109.5
C6—C5—H5A	120.1	H10B—C10—H10C	109.5
			
C8—N1—C1—C2	168.17 (8)	C4—C5—C6—C7	1.03 (16)
N1—C1—C2—C3	−138.75 (10)	C5—C6—C7—C2	−0.91 (15)
N1—C1—C2—C7	44.10 (12)	C3—C2—C7—C6	−0.02 (14)
C7—C2—C3—C4	0.83 (15)	C1—C2—C7—C6	177.09 (9)
C1—C2—C3—C4	−176.43 (9)	C1—N1—C8—C10	166.04 (8)
C2—C3—C4—C5	−0.71 (16)	C1—N1—C8—C9	−71.33 (11)
C3—C4—C5—C6	−0.22 (16)		

### Hydrogen-bond geometry (Å, °)

**Table:**

*D*—H···*A*	*D*—H	H···*A*	*D*···*A*	*D*—H···*A*
N1—H1···Cl1	0.853 (13)	2.288 (13)	3.1296 (9)	168.8 (11)
N1—H2···Cl1^i^	0.877 (14)	2.255 (14)	3.1257 (9)	171.9 (13)

Symmetry codes: (i) *x*+1/2, −*y*+1/2, *z*.
